# Supplementary material for: Non-volatile rippled-assisted optoelectronic array for all-day motion detection and recognition
Source: Nat Commun. 2024 Feb 22;15:1613. doi: 10.1038/s41467-024-46050-z (PMC10881999; doi:10.1038/s41467-024-46050-z)
Supplement: Supplementary file 1 — Supplementary Information [file 41467_2024_46050_MOESM1_ESM.pdf]

Supplementary information

Non-volatile rippled-assisted optoelectronic array for all-day motion detection and recognition

Xingchen Pang[1,§], Yang Wang[1,2,3,§]*, Yuyan, Zhu[1], Zhenhan Zhang[1], Du Xiang[4,5,6]*, Xun Ge[2], Haoqi Wu[1], Yongbo Jiang[1], Zizheng Liu[1], Xiaoxian Liu[1], Chunsen Liu[1,4], Weida Hu[2]*, Peng Zhou[1,3,4]*

[1]State Key Laboratory of ASIC and System, School of Microelectronics, Fudan University, Shanghai 200433, China

[2]State Key Laboratory of Infrared Physics, Shanghai Institute of Technical Physics, Chinese Academy of Sciences, Shanghai 200083, China

[3]Shanghai Frontiers Science Research Base of Intelligent Optoelectronics and Perception, Institute of Optoelectronics, Fudan University, Shanghai 200433, China

[4]State Key Laboratory of Integrated Chip and System, Frontier Institute of Chip and System, Fudan University, Shanghai 200433, China

[5]Zhangjiang Fudan International Innovation Center, Fudan University, Shanghai 200433, China

[6]Shanghai Qi Zhi Institute, Shanghai 200232, China

[§]These authors contributed equally: Xingchen Pang, Yang Wang

*email: yang_wang@fudan.edu.cn; xiang_du@fudan.edu.cn; wdhu@mail.sitp.ac.cn; pengzhou@fudan.edu.cn

Table of contents

Section 1. Characterization of the RAO processor 3

Section 2. Mechanism of mobility enhancement and first-principles calculation..8

Section 3. Response time and responsivity demonstration.....................................11

Section 4. Memory and PPC, NPC mechanisms of the RAO processor 13

Section 5. Linearity between photon responsiveness and drain voltage 16

Section 6. Wide spectrum responsiveness of the RAO processor 17

Section 7. Repetition stability of the RAO processor...21

Section 8. The RAO array demonstration 22

Section 9. Consistency between devices with mechanically exfoliated films and CVD films. 23

Section 10. Generality and flexibility of sr-SiN_x 26

Section 11. Contact resistance and its influence on mobility calculation. 32

Section 12. Methodology for motion detection and recognition 33

Section 13. Implementation in ANN 34

Section 14. Table of advanced optoelectronic devices 35

Reference 36

Section 1. Characterization of the RAO processor

Supplementary Fig. 1a shows the energy dispersive X-ray spectroscopy element mapping characterization of the device. The elements of Mo and S are strictly limited within the range of a single atom, proving the monolayer nature. The N and Si elements are within the range of sr-SiNx dielectric. Elements Cr and Au are the contact metal.

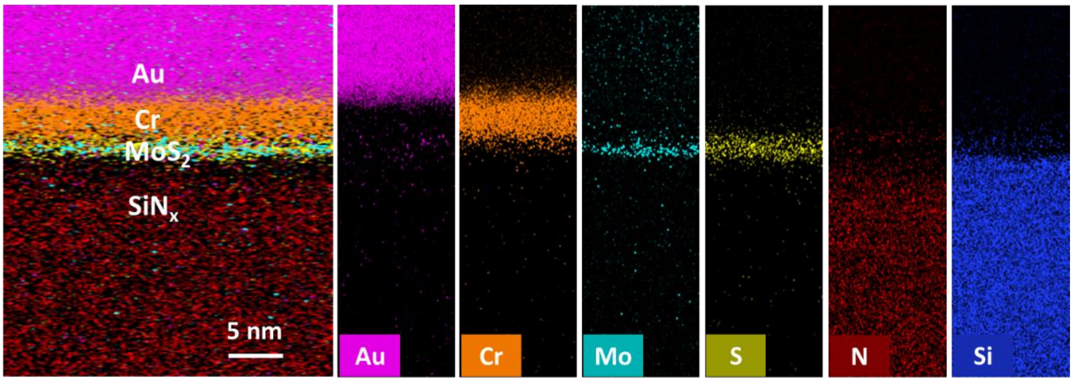

Supplementary Fig. 1 | Elements distribution of the RAO processor. The scanning transmission electron microscope and energy dispersive X-ray spectroscopy illustrate the monolayer property of the MoS_2. The mapping scale is 5 nm.

Supplementary Fig. 2a shows the surface of the specially treated sr-SiN_x measured by AFM. The average deviation and square deviation are 1.573 nm and 3.407 nm, respectively. **Supplementary Fig. 2b** shows the transfer curve of the RAO processor, which shows an obvious memory window in the anticlockwise direction. Also, we fabricated a transistor on the normal Si_3N_4 dielectric and measured the transfer characteristics, as shown in **Supplementary Fig. 2c-d**. The surface of the normal Si_3N_4 is measured by AFM and the deviation is much lower than that of the specially treated one. The transfer curve shows a negligible memory window, which is consistent with the previous works.

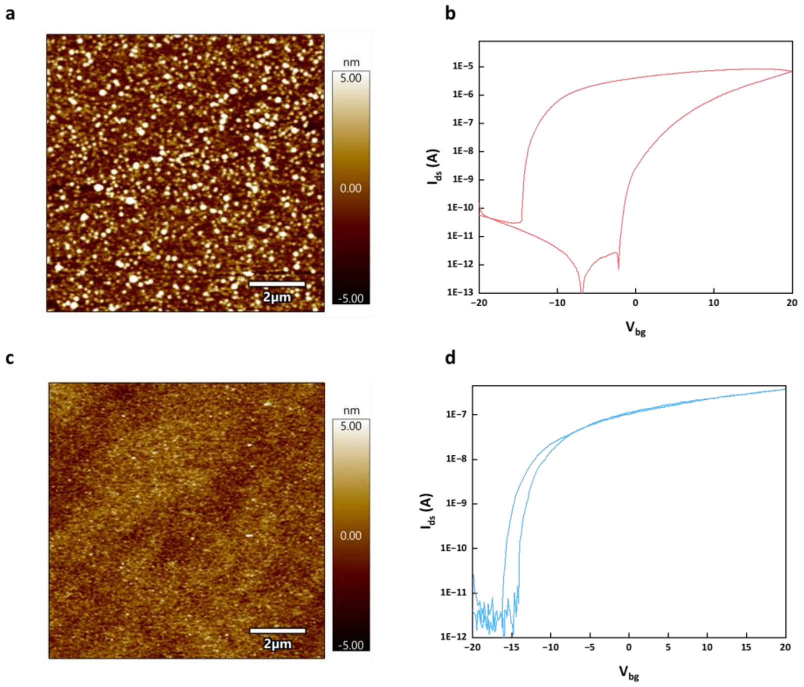

Supplementary Fig. 2 | Comparison between specially treated sr-SiN_x surface and normal SiN_x surface. a, surface of the specially treated sr-SiN_x measured by AFM. **b,** Transfer curve of the RAO processor. **c,** Surface of the normal SiN_x measured by AFM. **d,** Transfer curve of the MoS_2 device fabricated on the normal SiN_x dielectric.

Supplementary Fig. 3 shows the comparison of the photocurrent and dynamic range of the phototransistors on different substrates. Here, we define the difference between the maximum and minimum currents as the optical storage dynamic range of the all-in-one device. It is worth noting that the Dynamic Range of the device fabricated on the smoother surface is much smaller than that on the rougher counterpart. The mobility comparison of flat and rippled interface is shown in **Supplementary Fig. 4**. The wet polishing method is used to decrease the roughness of the sr-SiN_x substrate, which can be seen in **Supplementary Fig. 5a-c**.

Supplementary Fig. 6 shows the energy dispersive X-ray spectroscopy of the sr-SiN_x dielectric layer. We've tested 10 locations on the sr-SiN_x substrate and the results are shown in the **Supplementary Table. 1**. The average Si/N ratio is 1.397:1.

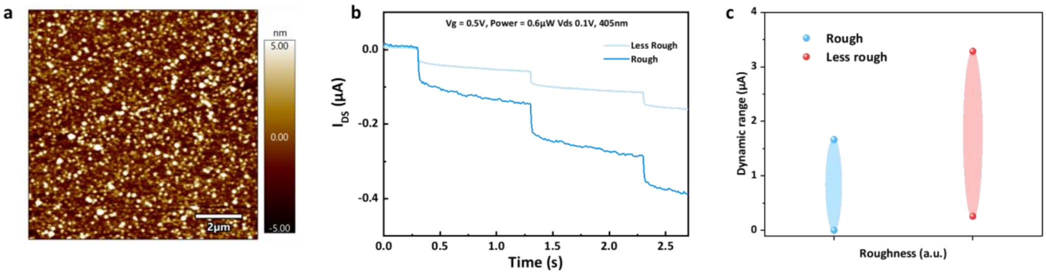

Supplementary Fig. 3 | Comparison of the photocurrent and dynamic range of the phototransistors on different substrates. a, Surface of the specially treated sr-SiN_x measured by AFM with smaller roughness. **b,** Comparison of the photocurrent with different roughnesses. **c,** Dynamic range of the all-in-one device with different roughnesses.

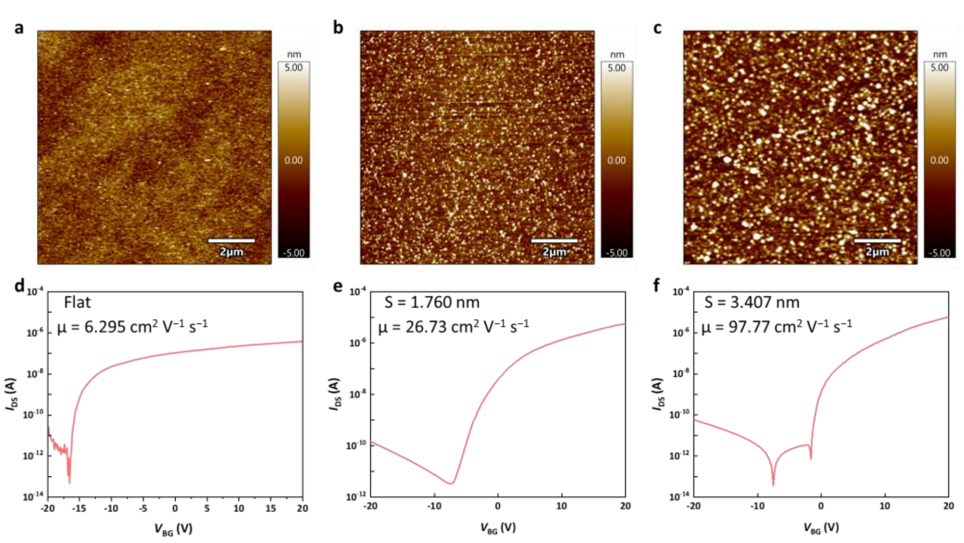

Supplementary Fig. 4 | Roughness and transfer curves of devices on both flat and rippled silicon nitride interface. a, Roughness of the device on the flat interface by AFM. b, Roughness of device on the rippled interface with less roughness by AFM. c, Roughness of device on the pristine rippled interface by AFM. d, The transfer curve and mobility of the device on the flat interface. The mobility is 6.295 cm^2 V^{-1} s^{-1}. e, The transfer curve and mobility of the device on the rippled interface with less roughness. The mobility is 26.73 cm^2 V^{-1} s^{-1} f, The transfer curve and mobility of the device on the pristine rippled interface. The mobility is 97.77 cm^2 V^{-1} s^{-1}, which shows a remarkable enhancement brought by the rippled interface.

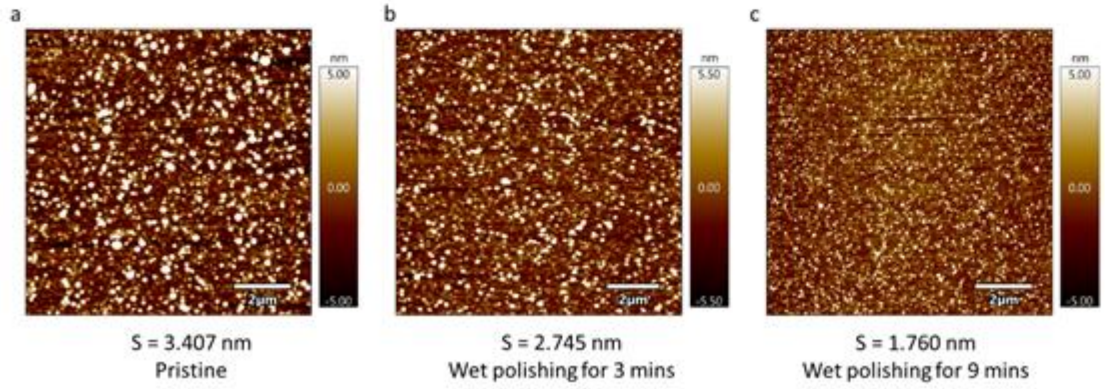

Supplementary Fig. 5 | Morphology of bulged SiN_x/Si substrates: AFM images of SiN_x/Si rippled substrate morphology with different roughness obtained using the wet polishing method. a, pristine surface of bulged SiN_x/Si substrates, of which the standard deviation of the roughness is 3.407 nm. b, Surface with process of wet polishing method (85 %wt phosphoric acid, 170 °C) for 3 minutes, of which the

standard deviation of the roughness is 2.745 nm. c, Surface with process of wet polishing method (85 %wt phosphoric acid, 170 °C) for 9 minutes, of which the standard deviation of the roughness is 1.760 nm.

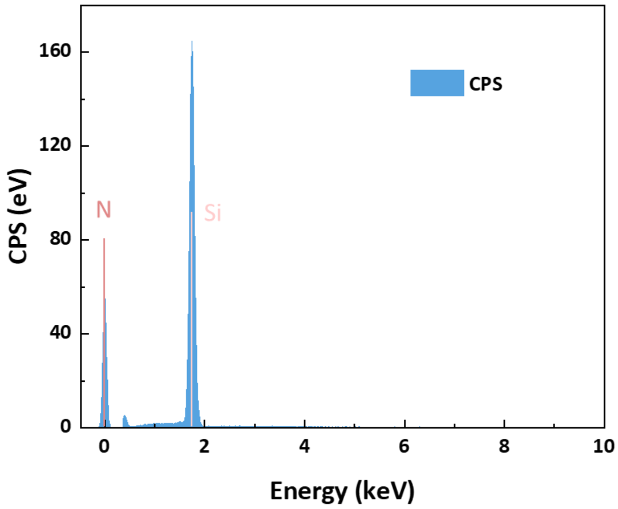

Supplementary Fig. 6 | Energy-dispersive X-ray spectroscopy of the sr-SiN_x layer. The first peak from the left represents the N element, and the second peak represents the Si element.

Position index	Si percentage (%)	N percentage (%)
**1**	57.98	42.02
**2**	57.60	42.40
**3**	58.90	41.10
**4**	58.43	41.57
**5**	58.35	41.65
**6**	59.59	40.41
**7**	57.38	42.62
**8**	59.40	40.60
**9**	58.08	41.92
**10**	57.14	42.86
**Average**	58.285	41.715

Supplementary Table 1 | Si and N element ratio at ten different positions of the sr-SiNx substrate in energy-dispersive X-ray spectroscopy.

Section 2. Mechanism of mobility enhancement and first-principles calculation.

In the Nature Electronics paper[1], lattice distortions can reduce electron–phonon scattering in 2D materials and thus improve the charge carrier mobility. The ripples in the MoS_2 caused by the bulged substrate lead to a change in the dielectric constant and a suppressed photon scattering, which leads to mobility enhancement.

Also, a first-principles calculation has been performed to obtain this mechanism of monolayer MoS_2. The calculation details are as follows:

Our first-principles calculations were performed with Vienna Ab-initio Simulation Package (VASP)[2, 3] using the projector augmented wave (PAW)[4] method. The exchange-correlation interactions were handled with Perdew-Burke-Ernzerhof (PBE)[5] functional. The cutoff energy for the plane-wave expansion was set to 400 eV in the whole process. The atom positions were relaxed until forces on them were less than 10^{-2} eV/Å. The rippled MoS_2 was constructed by sin function based on the $1 \times 3 \times 1$ supercell of orthogonal MoS_2. The rippled heights were selected 1, 2, and 3 Å, respectively, corresponding to the vertical distance between the highest and lowest Mo atoms. For the first Brillouin zone sampling, Γ-centered Monkhorst-Pack k-point meshes of $8\times2\times1$ and $16\times3\times1$ were used to perform structural relaxation and optical properties calculations. In addition, a vacuum layer larger than 15 Å was added to reduce the mirror interactions.

To elucidate the impact of substrate-induced bulges on the dielectric constant, we conducted dielectric function calculations for monolayer MoS_2 supercells employing four idealized models: flat MoS_2 and corrugated MoS_2 with curvature heights of 1 Å (Rippled-1 Å), 2 Å (Rippled-2 Å), and 3 Å (Rippled-3 Å). Progressing from flat MoS_2 to corrugated-1 Å, and from Rippled-1 Å to Rippled-3 Å, the relationship reveals an increasing curvature height, resulting in non-uniform strain within the twisted MoS_2. The curvature height is defined as the difference in height between the highest and lowest Mo atoms, as illustrated in **Supplementary Fig. 7a-d**. Energy band structure calculations for these four model structures are presented in **Supplementary Fig. 7e-h**, indicating progressively diminishing energy bands in MoS_2 as curvature increases.

To further investigate the relationship of the mobility of MoS_2, the mobility μ can be

obtained by the following[6]:

$$\mu = \frac{M_M M_X A t^2 \varepsilon^2 (\hbar\omega)^2}{16\pi^2 e^3 n m * Z_{MB}^2 (\sqrt{M_M} + \sqrt{M_X})^2} \tag{1}$$

where the M_M, and M_X are the atomic mass of metal and chalcogen atoms, A is the area of the unit cell, n is the Bose−Einstein distribution, t is the effective thickness, ε is the in-plane optical dielectric constant, Z_{MB} is the Born effective charge of the M. t and ε can be approximated by the bulk dielectric constant and the interlayer distance in the bulk material. In accordance with Equation (1), it is observed that the material's mobility is directly proportional to ε^2. The calculations of the real part of the dielectric function (ε1) for the aforementioned four model structures are presented in **Supplementary Fig. 8** and **Supplementary Fig. 9**, where the static permittivity of MoS_2 is determined by the value at $E \to 0$. It is noteworthy that these calculations exclude the ionic contribution, as its impact is negligible in comparison to the electronic contribution. The results demonstrate that both the in-plane and out-of-plane dielectric constants of MoS_2 exhibit an increase with the height of curvature. Consequently, the ripples in the MoS_2 caused by the bulged substrate result in an increase in the dielectric constant, This, in turn, contributes to the enhancement of mobility.

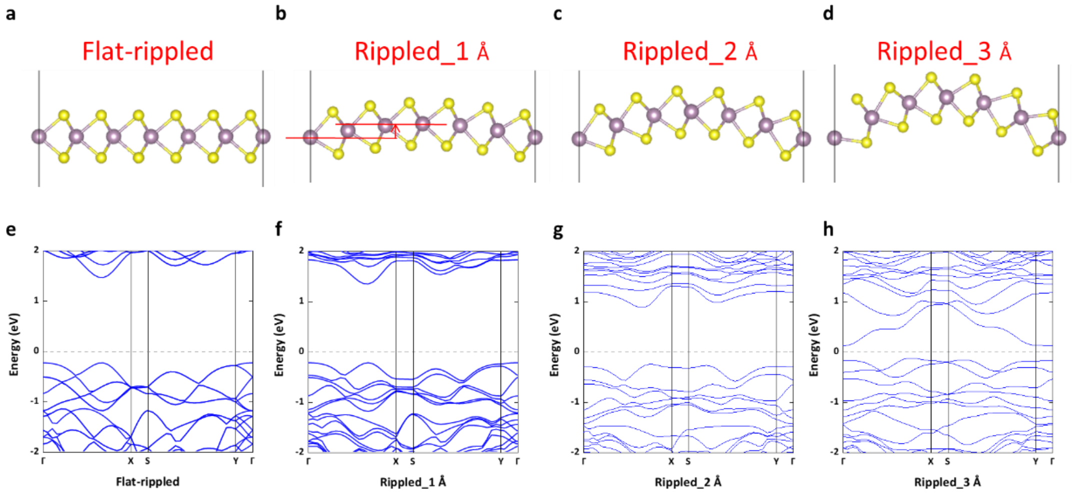

Supplementary Fig. 7 | 3D views and electronic band structures of rippled MoS_2 crystal from different curvature height. a-d, 3D views of (a) flat-MoS_2 and rippled-MoS_2 with (b) 1 Å, (c) 2 Å and (d) 3 Å curvature heights. e-h, Electronic band structures of (e) flat-MoS_2 and rippled-MoS_2 with (f) 1 Å, (g) 2 Å and (h) 3 Å curvature heights.

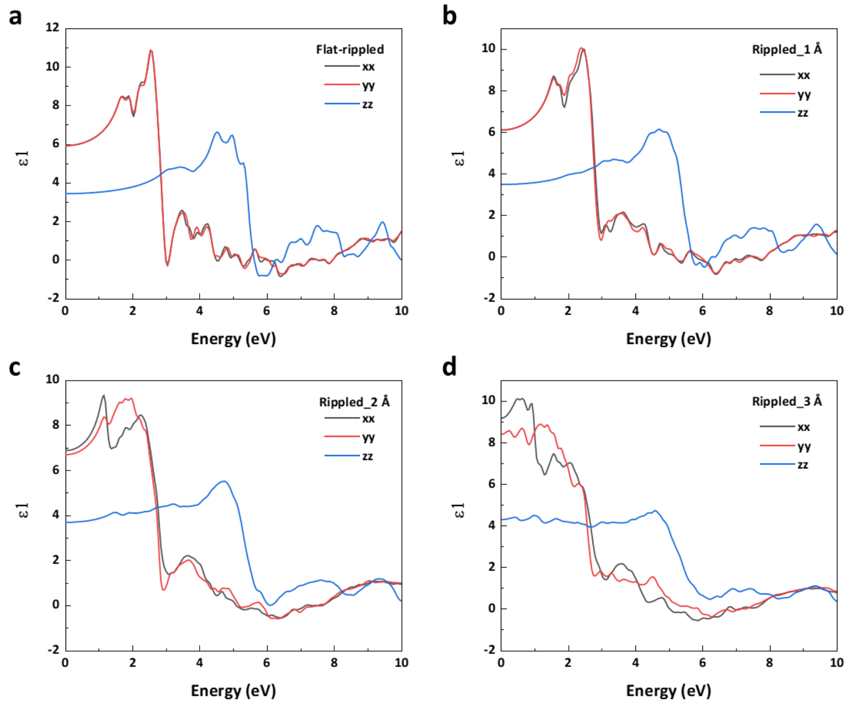

Supplementary Fig. 8 | First-principles calculations of the dielectric function's real part (ε1) for monolayer MoS_2. a-d, Flat-MoS_2 and rippled-MoS_2 with curvature heights of 1 Å, 2 Å, and 3 Å.

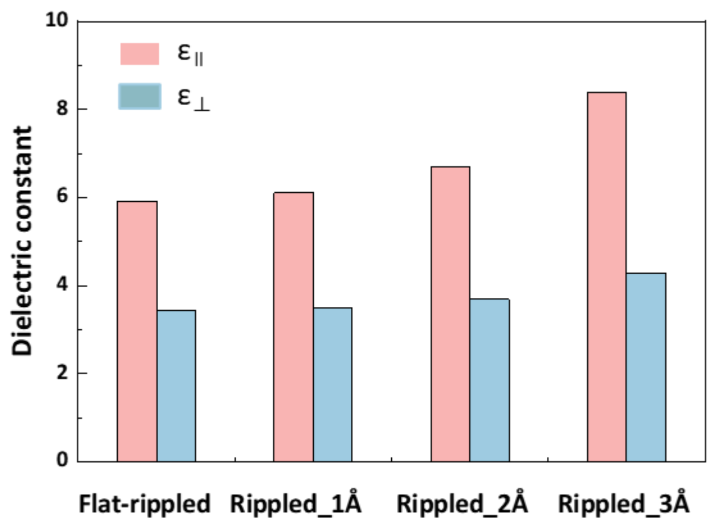

Supplementary Fig. 9 | Comparison of the static dielectric constants of monolayers of MoS_2 with varying curvature heights (flat-rippled, rippled-1 Å, rippled-2 Å, and rippled-3 Å), calculated based on first principles. $\varepsilon_{//}$ and $\varepsilon_{\perp}$ represent in-plane and out-of-plane dielectric constants of MoS_2, respectively.

Section 3. Response time and responsivity demonstration

The RAO device is composed of the monolayer MoS_2, as shown in Fig. 2a, the response time of the RAO device is mainly affected by the mobility and the channel length of the device. As we use the rippled interface of silicon-rich silicon nitride, the response time has been highly reduced. The photocurrent with the light stimuli lasting in the range from 1 ms to 50 ms is shown in **Supplementary Fig. 10**.

The responsivity data and its incident power dependence in both visible and infrared regions. **Supplementary Fig. 11a** illustrates the responsivity under varying incident power at a wavelength of 405 nm, while **Supplementary Fig. 11b** depicts the responsivity of the first state under varying incident power at a wavelength of 940 nm. Notably, as the power increases, the responsivity decreases. Additionally, **Supplementary Fig. 11c** showcases the responsivity with gate voltage ranging from 0 V to 15 V, with the maximum responsivity recorded as 181.66 A/W at a gate voltage of 6.6 V.

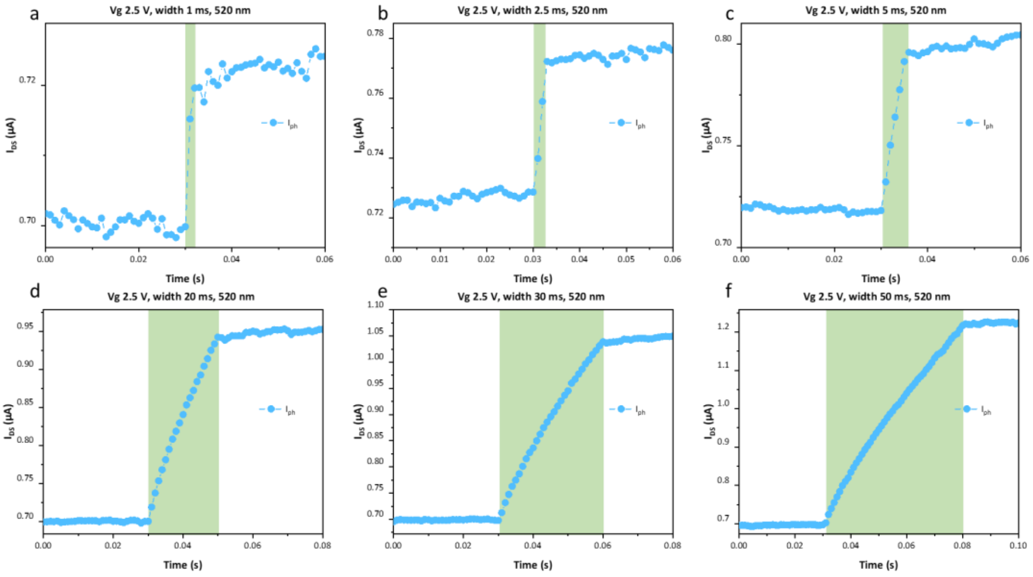

Supplementary Fig. 10 | The IDS with different duration of stimuli from 1 ms to 50 ms. The durations are selected as **(a)** 1 ms, **(b)** 2.5 ms, **(c)** 5 ms, **(d)** 20 ms, **(e)** 30 ms, **(f)** 50 ms, respectively.

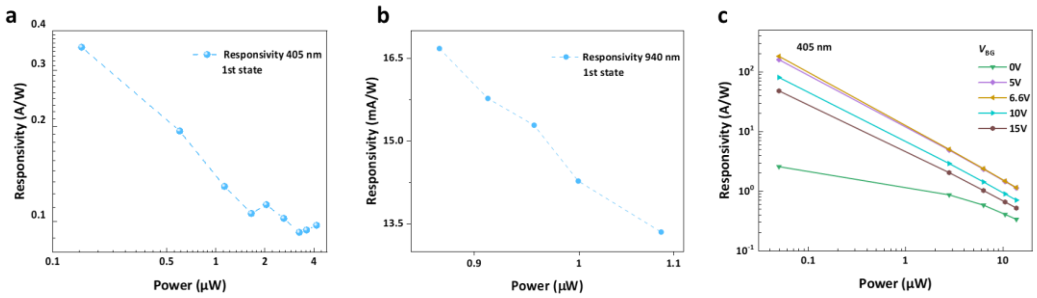

Supplementary Fig. 11 | The responsivity depends on multiple parameters. The responsivity of the first state under varying incident power at the wavelength of **(a)** 405 nm and **(b)** 940 nm, respectively. c, The responsivity of the device with different gate voltages at the wavelength of 405 nm.

Section 4. Memory and PPC, NPC mechanisms of the RAO processor

The silicon-rich SiN_x dielectric layer contains numerous natural defects that are capable of trapping holes. It is worth mentioning that heavily p-doped silicon functions as a natural hole reservoir and can provide a sufficient number of holes for injection. During the writing process, a +20 V gate voltage is applied to drive hole injection from the heavily p-doped silicon to the sr-SiN_x dielectric. The injected holes are subsequently trapped by hole trapping centers, which act as positive local gates that enhance the electron concentration and channel conductance, thereby corresponding to the memory of the low resistance state, which is shown in **Supplementary Fig. 12a-b**. For the light erase process, a fixed -3 V gate voltage is applied accompanied by the optical stimulus, in which the holes in the dielectric are released. The reduction of stored holes leads to a smaller channel current, resulting in negative photo conductance (NPC). The carrier distribution and band diagram of NPC are shown in **Supplementary Fig. 12c**.

During the erasing process, a fixed -20 V gate voltage is applied to release the trapped holes, leading to a decrease in channel conductance that corresponds to the memory of the high resistance state (shown in **Supplementary Fig. 12d-e**). As for the light write process, a fixed 3 V gate voltage is applied accompanied by the optical stimulus, in which the holes in the dielectric are trapped as the photon carriers generated by optical stimuli overcome the MoS_2/sr-SiN_x interface barrier (shown in **Supplementary Fig. 12f**), leading to the positive photo conductance (PPC).

The retention of the RAO processor is shown in **Supplementary Fig. 13**. Also, we've measured the output curves of different states tuned by light stimuli, as shown in **Supplementary Fig. 14**.

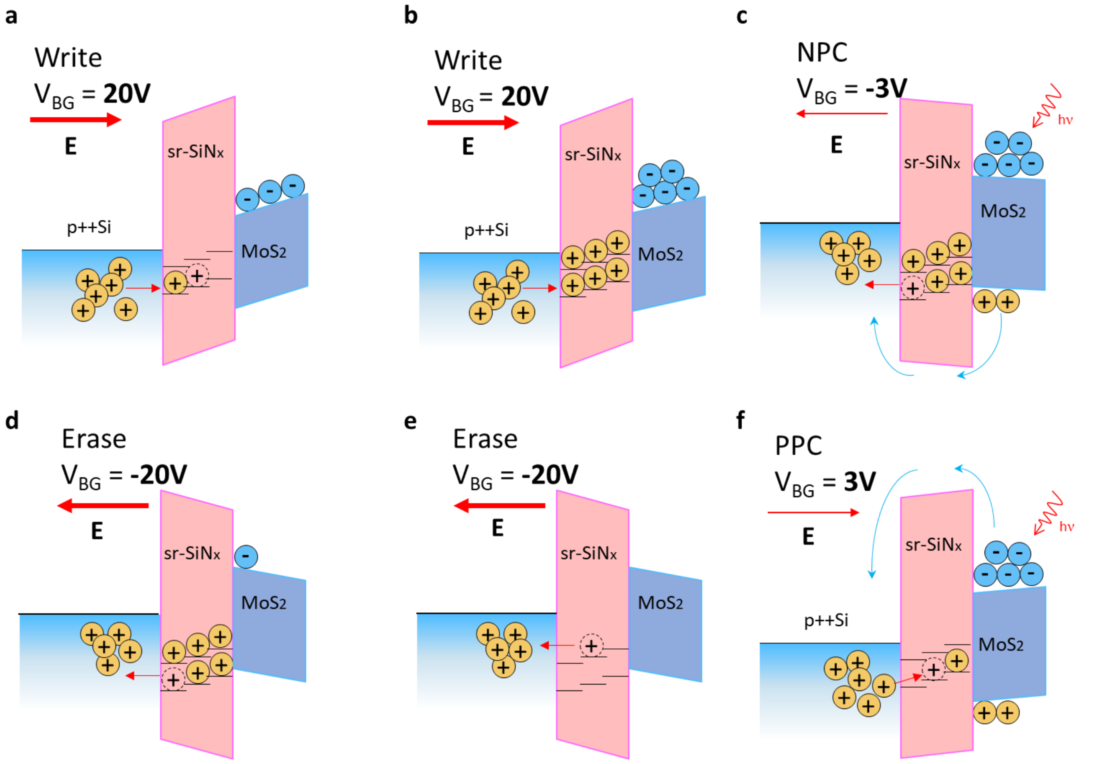

Supplementary Fig. 12 | Positive and negative photoconductive mechanisms. a-b, The writing process causes the device to transition to a low-resistance state. **c,** Light erase process and negative photo conductance. **d-e,** The erasing process makes the device return to a high resistance state. **f,** Light write process and positive photo conductance.

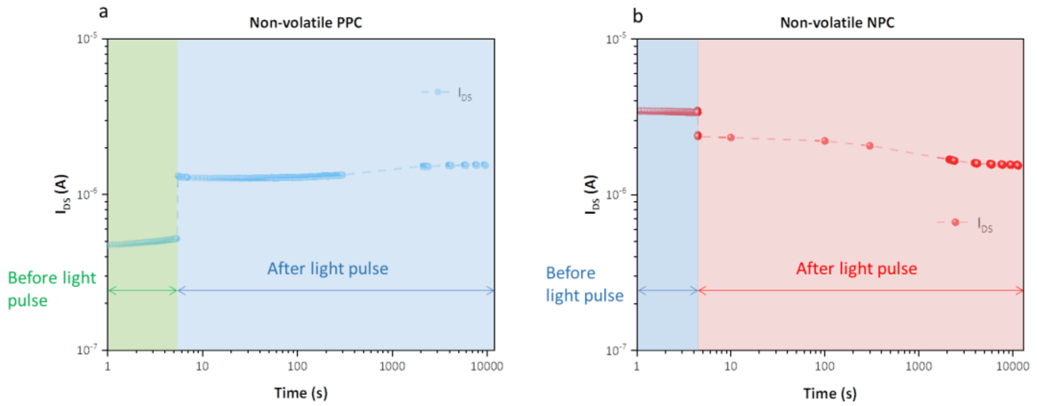

Supplementary Fig. 13 | Persistent retention characteristics of PPC and NPC. a, The retention of PPC is demonstrated, the shallow green region represents the drain current before the light stimuli, the shallow blue region represents the drain current after the light stimuli. **b,** The retention of NPC is demonstrated.

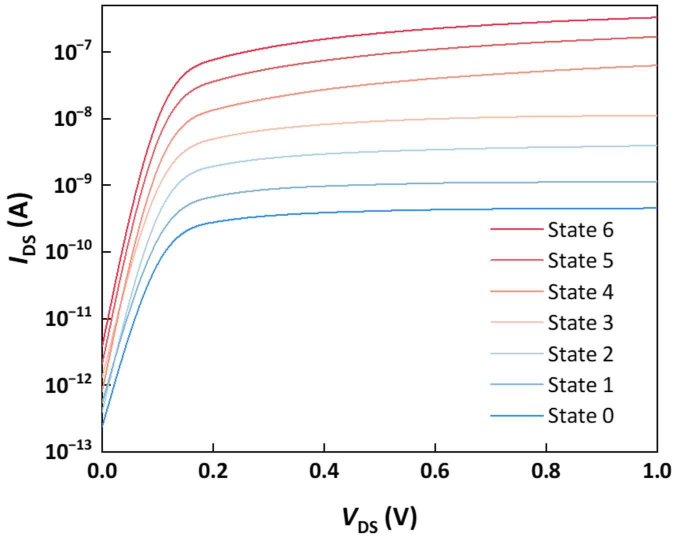

Supplementary Fig. 14 | They were generated through light stimuli tuning at V_{BG} = 0 V. The device states were modulated by 10 ms light stimuli at the wavelength of 520 nm.

Section 5. Linearity between photon responsiveness and drain voltage

Supplementary Fig. 15a-b illustrates the positive and negative photon responses to light stimuli at varying drain voltages. It is evident that increasing drain voltage results in an increase in the magnitude of the photocurrent. Additionally, we extracted the NV-PPC and NV-NPC, both exhibiting linearity with respect to drain voltage, as demonstrated in **Supplementary Fig. 15c**.

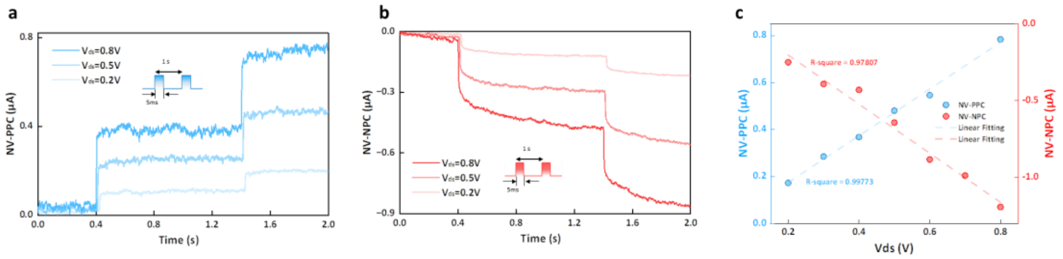

Supplementary Fig. 15 | Linearity between photoresponsive and drain voltage. a-b, The NV-PPC and NV-NPC responses were measured at various drain voltages during continuous light pulse stimulation. **c,** The NV-PPC and NV-NPC were fitted using linear regression.

Section 6. Wide spectrum responsiveness of the RAO processor

To evaluate the device's capability to operate continuously under varying laser power and wavelengths, we measured the NV-PPC and NV-NPC responses at different wavelengths (ranging from visible to near-infrared) and laser power, with up to 15 states. **Supplementary Fig. 16a-c** shows the time dependent current curve under different wavelengths of 405nm, 520nm and 940nm (near infrared). We also extracted the NV photocurrent for each stimulus and observed the linearity between the photocurrent and pulse number, as shown in **Supplementary Fig. 16d-f**.

Supplementary Fig 17-19 shows the 2D color mapping of NV photocurrent with different pulse number, laser power and wavelength. It is obvious that as the pulse number or laser power increases, the photocurrent also increases proportionally.

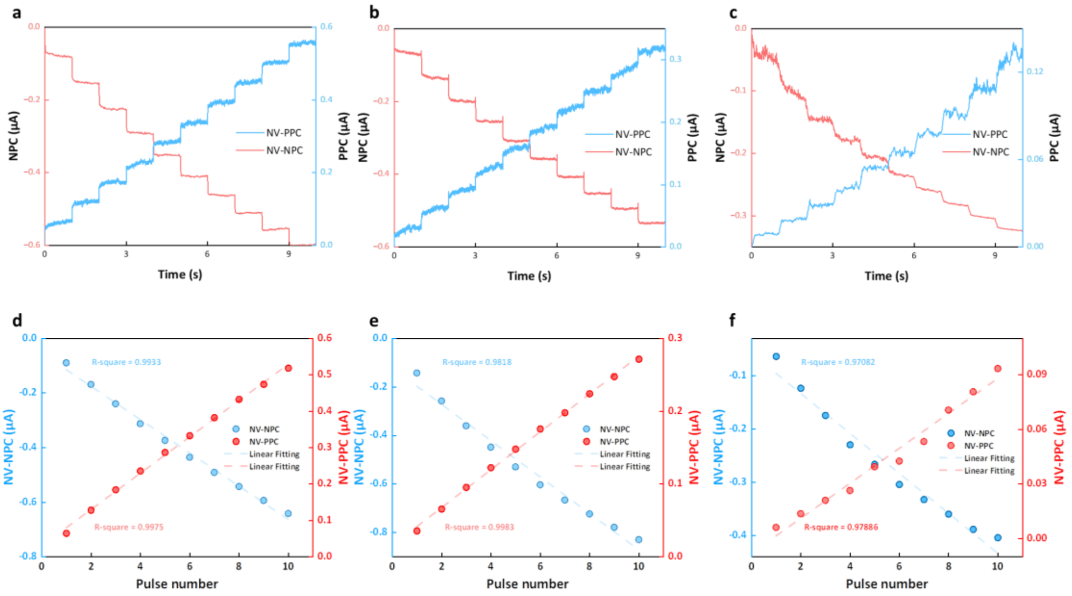

Supplementary Fig. 16 | Photo response under different wavelengths. a-c, Current curves are obtained under continuous light pulses with wavelengths of 405 nm, 638 nm, and 940 nm respectively. **d-f,** The NV-PPC and NV-NPC are extracted from the current curves and subjected to linear regression analysis for the wavelengths of 405 nm, 638 nm, and 940 nm respectively.

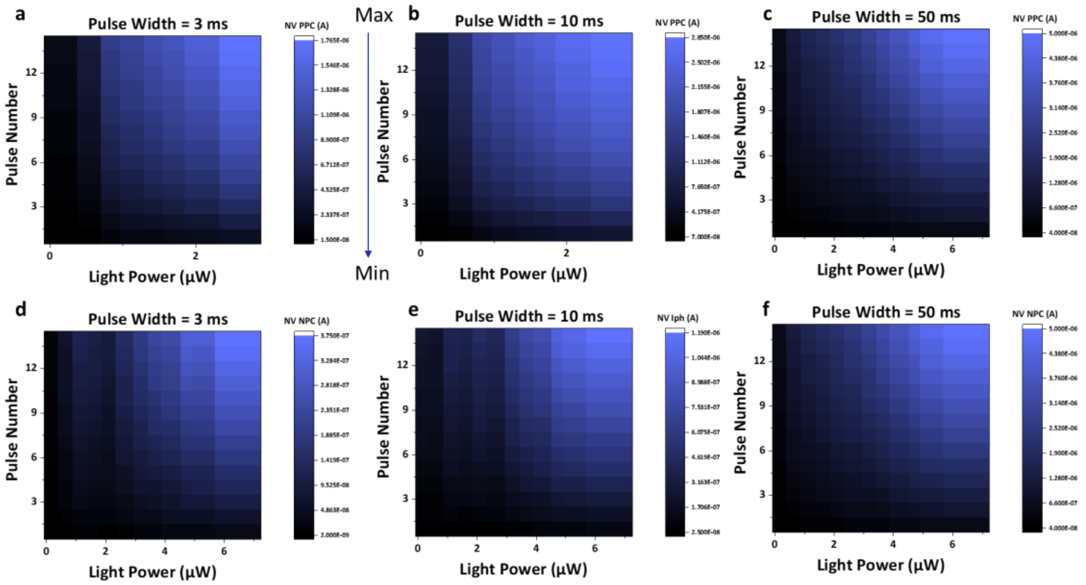

Supplementary Fig. 17 | NV-PPC and NV-NPC of the RAO processor under different pulse numbers and laser power at a wavelength of 405 nm. a-c, The NV-PPC was measured under varying pulse numbers and laser power. **d-f,** The NV -NPC was measured under varying pulse numbers and laser power.

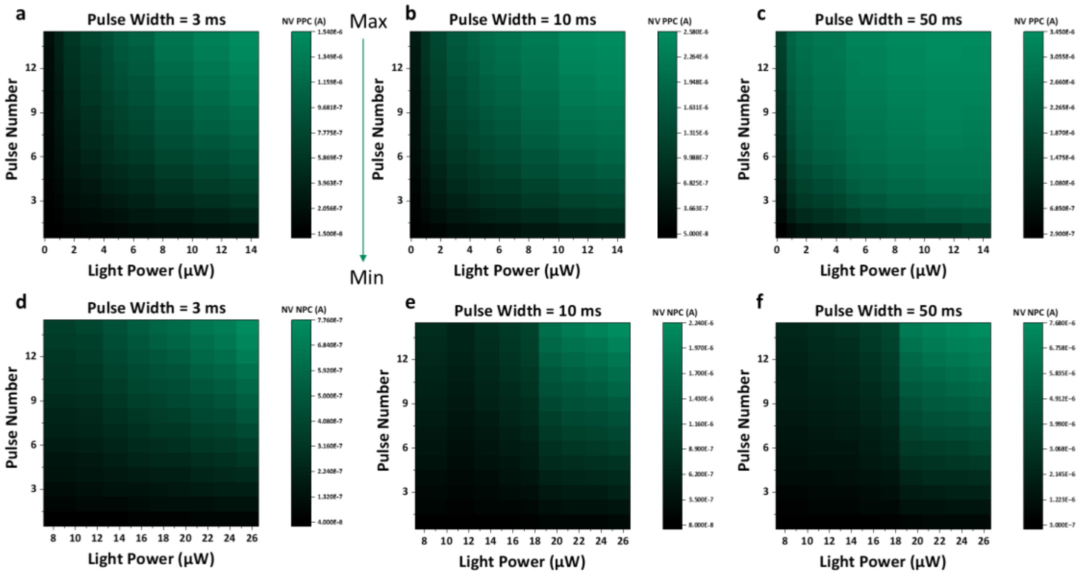

Supplementary Fig. 18 | NV-PPC and NV-NPC of the RAO processor under different pulse numbers and laser power at a wavelength of 520 nm. a-c, The NV-PPC was measured under varying pulse numbers and laser power. **d-f,** The NV -NPC was measured under varying pulse numbers and laser power.

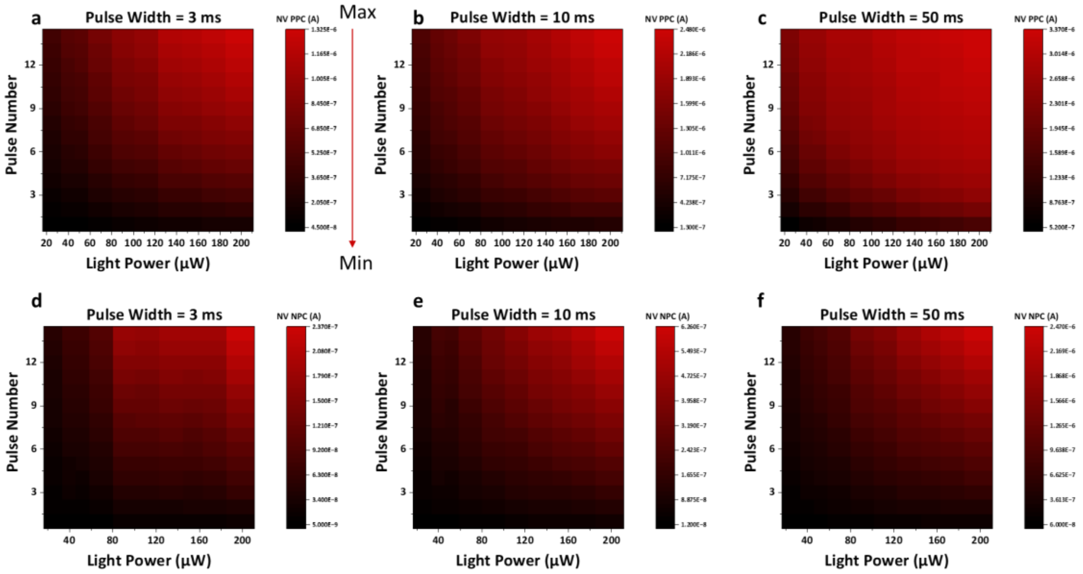

Supplementary Fig. 19 | NV-PPC and NV-NPC of the RAO processor under different pulse numbers and laser power at a wavelength of 638 nm. a-c, The NV-PPC was measured under varying pulse numbers and laser power. **d-f,** The NV -NPC was measured under varying pulse numbers and laser power.

Section 7. Repetition stability of the RAO processor

A cyclic test consisting of 50 cycles of positive and negative photoconductive operation (PPC and NPC) has been performed to confirm the stability of the RAO processor. **Supplementary Fig. 20a** shows the measured current curve of all 50 cycles. The dashed boxes correspond to the 4th cycle and the 49th cycle, respectively. **Supplementary Fig. 20b-c** shows the PPC and NPC of the 4th cycle under continuously light stimuli, respectively. **Supplementary Fig. 20d-e** shows the current curve of the 49th cycle using the same test configuration as all other cycles. Each cycle includes at least 10 states of NV photoconductance for both PPC and NPC under continuous light pulse stimulation. The repetition of PPC and NPC shows the stability of the RAO processor.

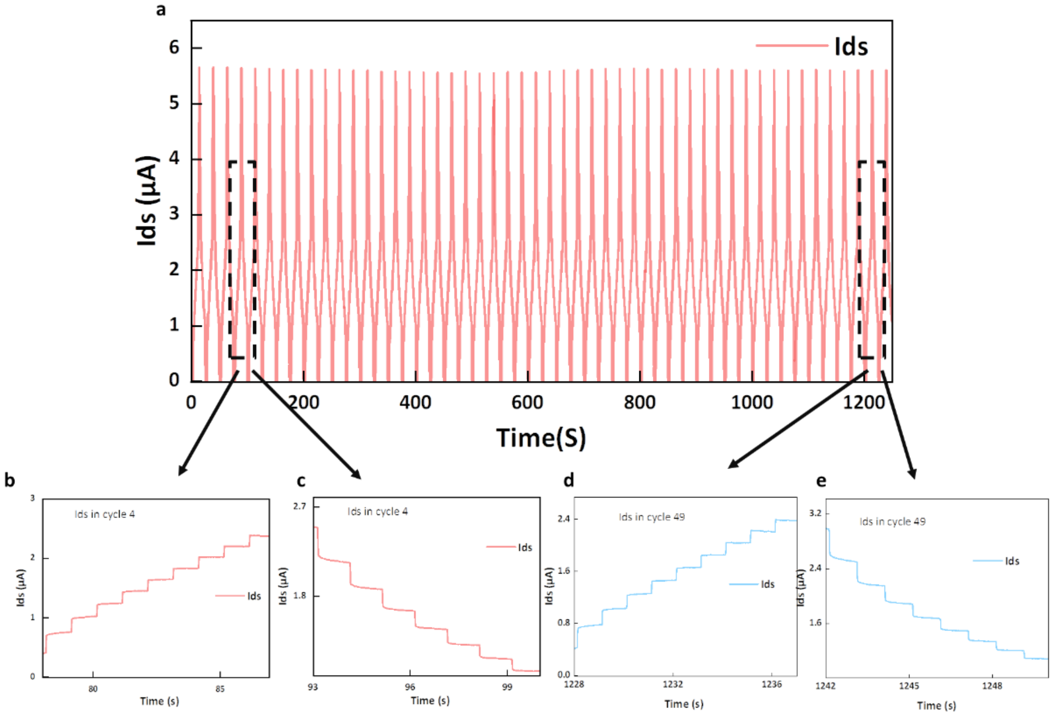

Supplementary Fig. 20 | 50 cycles of repetition for stability test of the RAO processor. a, Current curve of the stable test lasting around 1200 s. **b-c,** Current curves of PPC and NPC for the 4th cycle, respectively. **d-e,** Current curves of PPC and NPC for the 49th cycle, respectively.

Section 8. The RAO array demonstration

The detailed array fabrication process can be seen in Method of the manuscript. The microscope view of the RAO array is shown in **Supplementary Fig. 21a-c**, where the gold part represents the source and drain contact. The dashed box in the **Supplementary Fig. 21b** shows the channel region of MoS_2 monolayer. The SEM view of the RAO array is shown in **Supplementary Fig. 21d-f.** As shown in **Supplementary Fig. 21f,** the orange dashed box represents one of the SiO_2 region, the blue dashed box represents the channel region, the green dashed box represents part of the metal contact.

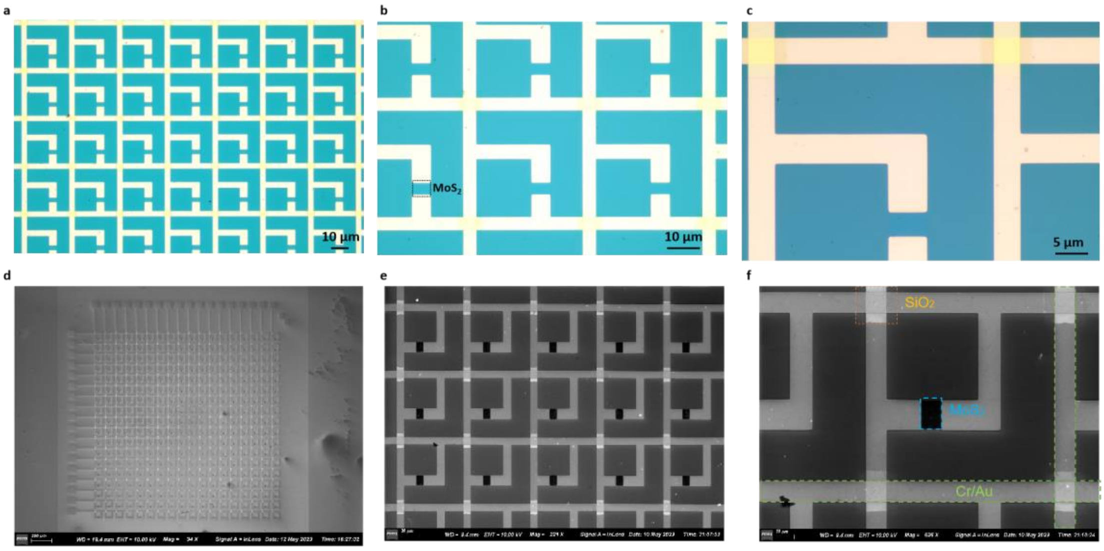

Supplementary Fig. 21 | Optical images of the RAO array at different magnifications. a, Enlarged microscope view of the 18×18 RAO array by 200x. **b,** Enlarged microscope view of the 6 RAO devices by 500x. **c,** More enlarged microscope view of single RAO device by 1000x. **d,** SEM view of the 18×18 RAO array. **e,** Enlarged SEM view of the 18×18 RAO array magnified by 221x. **f,** Enlarged SEM view of the 18×18 RAO array magnified by 626x.

Section 9. Consistency between devices with mechanically exfoliated films and CVD films.

(1). **Consistency in electrical properties:** The measurements on both ME and CVD devices showed a high level of consistency in electrical properties, as depicted in **Supplementary Fig. 22**. The measured mobilities of 97.77 $cm^2V^{-1}s^{-1}$ for CVD MoS_2 and 89.84 $cm^2V^{-1}s^{-1}$ for ME MoS_2 provide quantitative insights into the charge carrier mobility of these materials. The small variation in mobility suggests that both synthesis methods yield devices with similar charge transport characteristics. The overlapping memory window and a relatively small variation in mobility (9%) between CVD and ME devices underscore the comparable electrical performance of the two synthesis methods.

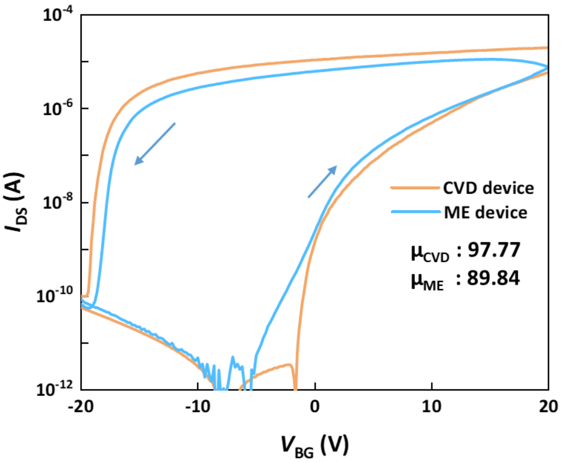

Supplementary Fig. 22 | Transfer curves and mobility of monolayer MoS_2 transistors by both CVD and ME. The transistors show a great consistency in electrical properties like memory and mobility. The mobility for device of CVD MoS_2 is 97.77 $cm^2V^{-1}s^{-1}$ and 89.84 $cm^2V^{-1}s^{-1}$ for the one by mechanically exfoliated film, of which the variation is less than 9%.

(2). **Consistency in optoelectronic characteristics:** The observation of a common trend in both CVD and ME devices, where responsivity decreases as effective incident light power increases (**Supplementary Fig. 23a-b**), indicates a consistent behavior in the optoelectronic response of MoS_2 devices. Also, in terms of non-volatile photocurrent, both devices exhibit a similarity under identical test conditions for both positive and negative photocurrent, as illustrated in **Supplementary Fig. 24a-b**. The similarity in optoelectronic responses between CVD and ME devices further emphasizes the comparable performance of these devices in terms of their behavior under varying light power conditions and continuous light stimuli.

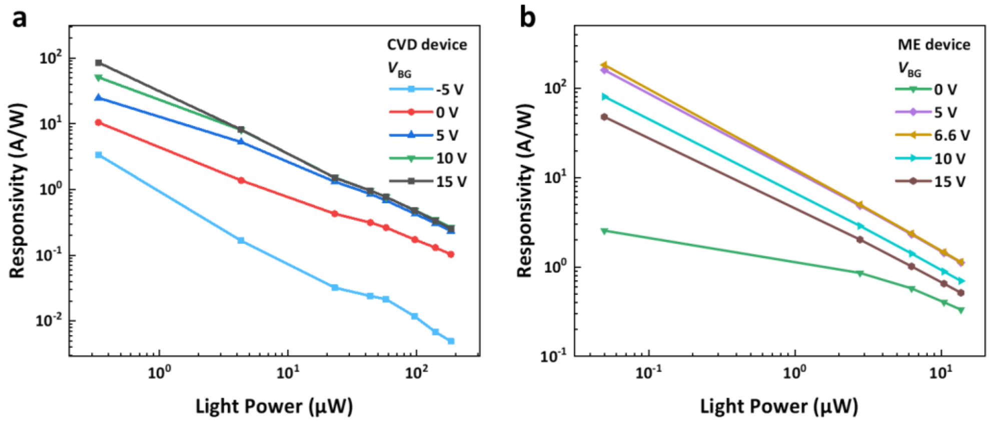

Supplementary Fig. 23 | Responsivity of both CVD and ME devices. a, The responsivity of the CVD device with different gate voltages at a wavelength of 405 nm. As the light power increments, the responsivity decreases. b, The responsivity of the ME device with different gate voltages at a wavelength of 405 nm. It shows the same trend as the CVD device when incident power increases.

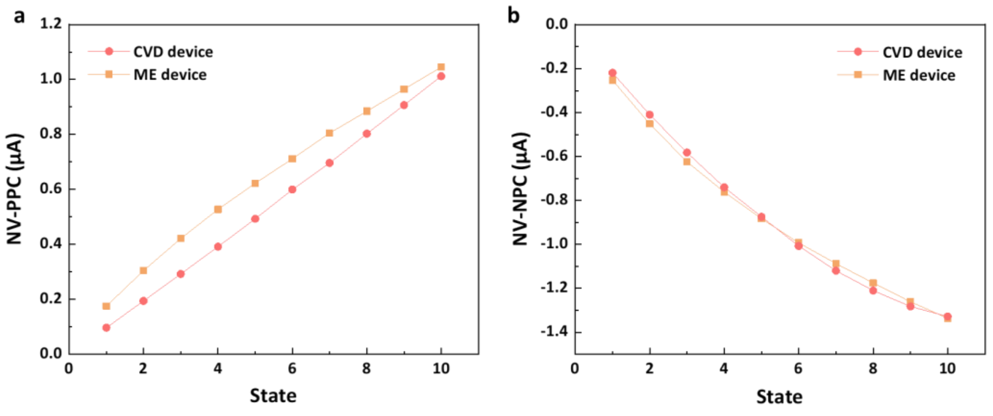

Supplementary Fig. 24 | Non-volatile positive and negative photocurrent of CVD and ME devices. a, Non-volatile positive photocurrent of CVD and ME devices with continuous light stimuli. b, Non-volatile negative photocurrent of CVD and ME devices with continuous light stimuli, where the incident power is constant 13.5 μW and the lasting time of stimuli is 20 ms at the wavelength of 520 nm.

Section 10. Generality and flexibility of sr-SiN_x

To further verify the functionality of the specially treated sr-SiN_x, we fabricated a phototransistor based on $MoTe_2$ on the same specially treated sr-SiN_x dielectric. We've tested the property of $MoTe_2$ to better evaluate the enhancement induced by the rippled interface. This result shows the rippled sr-SiN_x dielectric layer has good generality and flexibility for providing the functionality of both positive and negative photo response and memory capacity for some types of Transition Metal Dichalcogenides (TMD).

(1). **Memory capacity.** The observation of a memory gap in the transfer curve represents the memory capacity brought by the sr-SiN_x dielectric. Importantly, both MoS_2 and $MoTe_2$ devices over this specifically treated dielectric exhibit a remarkable memory gap (**Supplementary Fig. 25a-b**). It is also noteworthy that, owing to the ambipolar feature of $MoTe_2$, the transfer curve of the $MoTe_2$ device displays an additional, smaller memory gap. This underscores the versatility of the sr-SiN_x layer in imparting memory functionality.

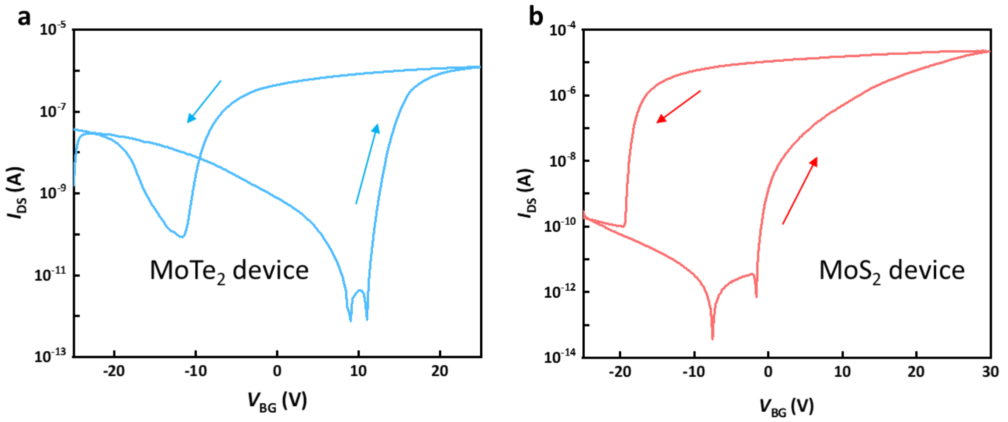

Supplementary Fig. 25 | Transfer curves of $MoTe_2$ and MoS_2 device. a, Transfer curve of $MoTe_2$ device at the V_{DS} = 0.1 V, where a huge memory gap and a smaller memory gap are observed. b, Transfer curve of MoS_2 device at the V_{DS} = 0.1 V, where a huge memory gap is observed.

(2). **Positive and negative photo response.** As shown in **Supplementary Fig. 25a-b**, the MoS_2 device shows the features of both positive photon response (PPC) and negative photo response (NPC) in the microamp scale. Similarly, the $MoTe_2$ device shows the same features of PPC and NPC in the scale of nanoamp, which is illustrated in **Supplementary Fig. 26**c-d. This indicates the generality of this sr-SiNx layer for providing the functionality of positive and negative photo response.

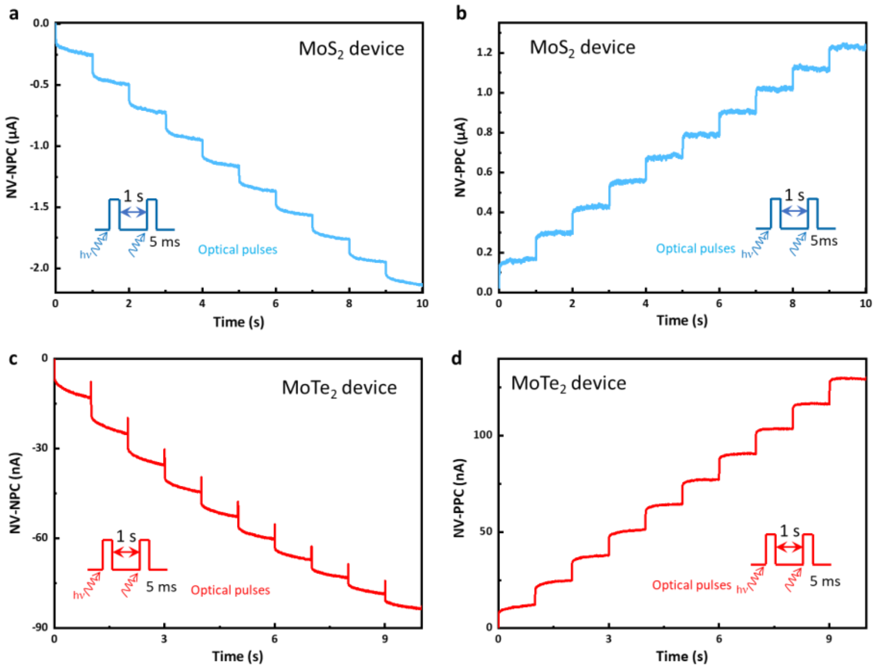

Supplementary Fig. 26 | Positive and negative photo response for both MoS_2 and $MoTe_2$ devices. a, Cumulative non-volatile positive photocurrent of MoS_2 device with progressive multilevel states under periodic optical pulses. b, Cumulative non-volatile negative photocurrent of MoS_2 device with progressive multilevel states under periodic optical pulses. c, Cumulative non-volatile negative photocurrent of $MoTe_2$ device with progressive multilevel states under periodic optical pulses. d, Cumulative non-volatile positive photocurrent of $MoTe_2$ device with progressive multilevel states under periodic optical pulses. For a-d, the lasting time is 5 ms and the period is 1 s at the wavelength of 520 nm. All devices use mechanically exfoliated films.

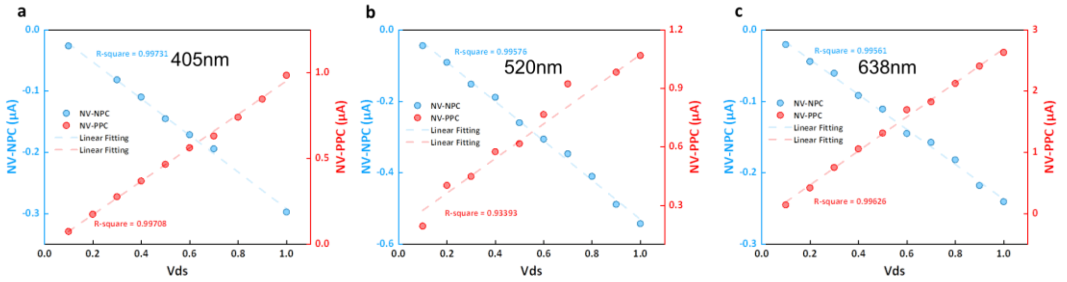

Supplementary Fig. 27 | Linearity of the rippled $MoTe_2$ processor with different wavelengths of 405 nm, 520 nm and 638 nm. a-c, Linearity with the various drain voltages under different wavelengths.

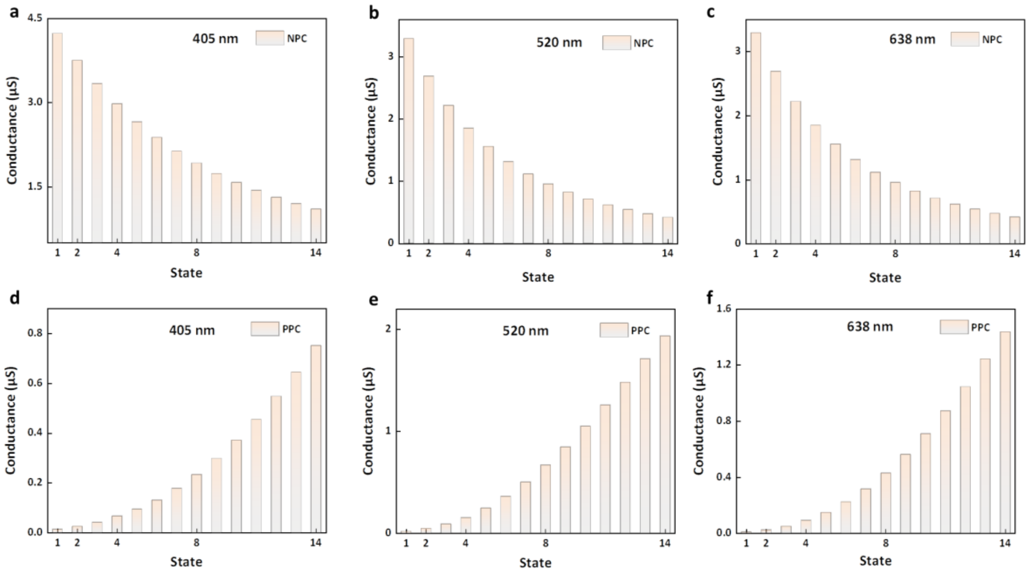

Supplementary Fig. 28 | Conductance states of both NV-PPC and NV-NPC by the same light stimuli under different wavelengths of 405 nm, 520 nm and 638 nm. a-c, State conductance of NV-NPC. **d-f,** State conductance of NV-PPC.

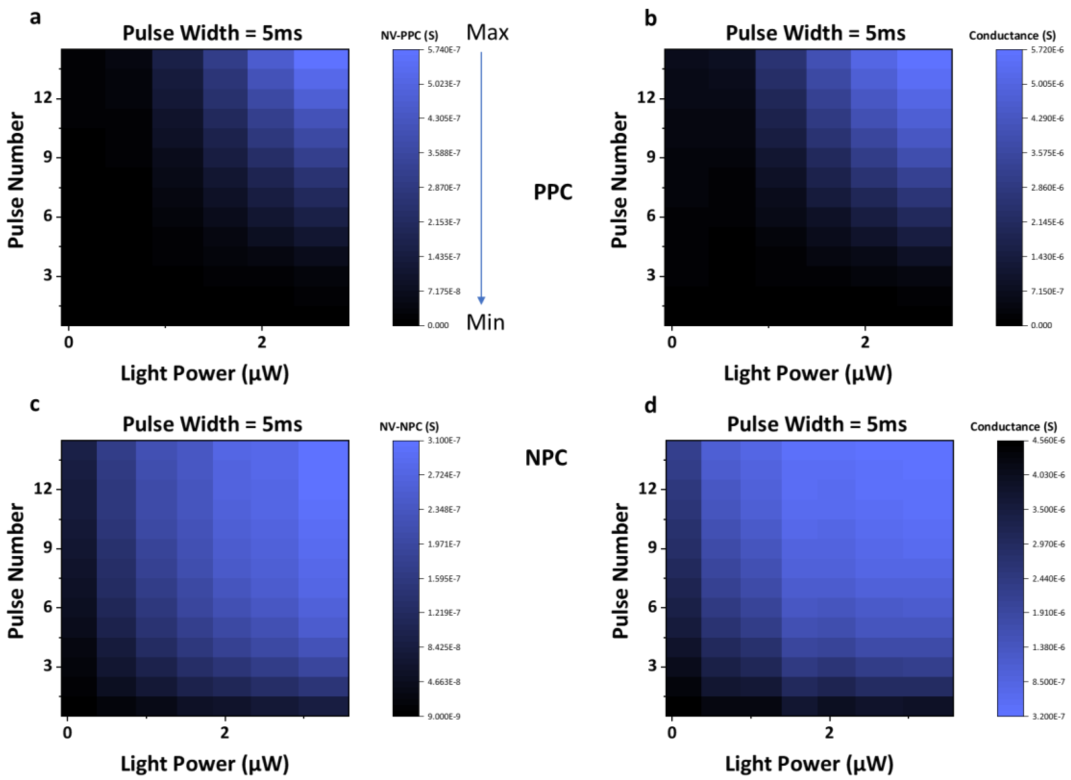

Supplementary Fig. 29 | NV-PPC and NV-NPC of the rippled $MoTe_2$ processor under different pulse number and laser power at wavelength of 405 nm. a-b, NV-PPC under different pulse number and laser power. **c-d,** NV-NPC under different pulse number and laser power.

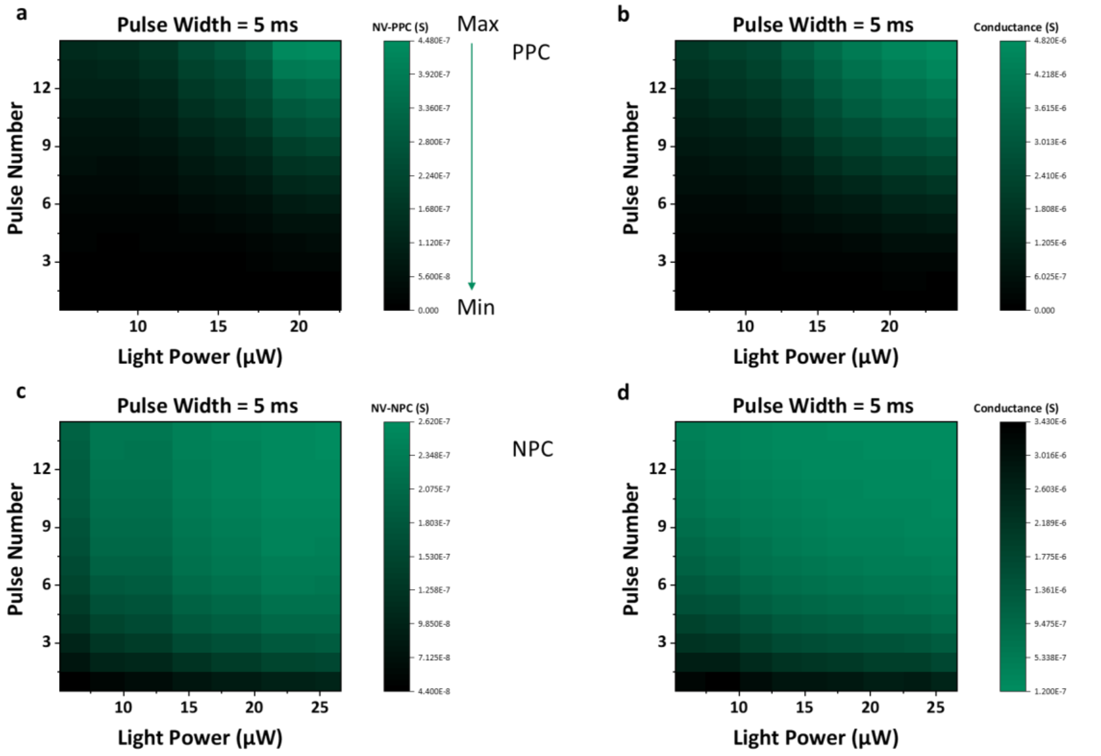

Supplementary Fig. 30 | NV-PPC and NV-NPC of the rippled $MoTe_2$ processor under different pulse number and laser power at wavelength of 520 nm. a-b, NV-PPC under different pulse number and laser power. **c-d,** NV -NPC under different pulse number and laser power.

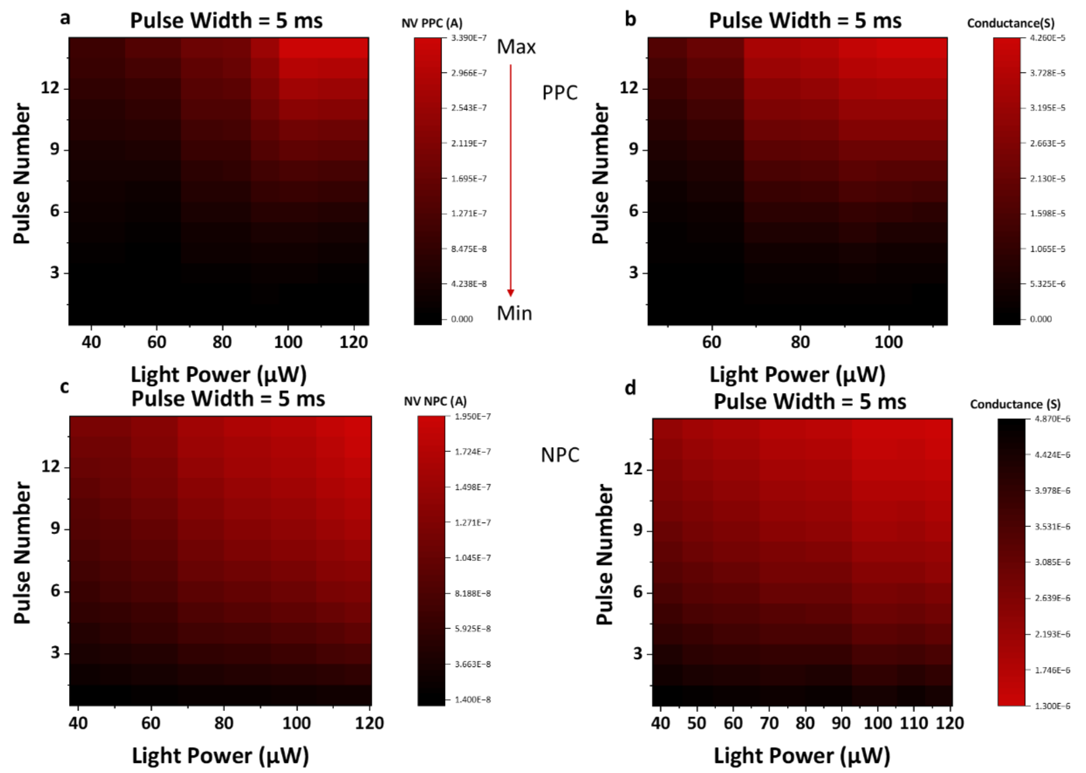

Supplementary Fig. 31 | NV-PPC and NV-NPC of the rippled $MoTe_2$ processor under different pulse numbers and laser power at a wavelength of 638 nm. a-b, NV-PPC under different pulse numbers and laser power. **c-d,** NV-NPC under different pulse numbers and laser power.

Section 11. Contact resistance and its influence on mobility calculation.

To isolate the effect of the contact resistance and the channel resistance, we employed the Transmission Line Model (TLM), aiming to determine the specific contact resistivity of a metal-semiconductor junction. RAO devices were fabricated with varying channel lengths, ranging from 6 μm to 14 μm, over a control sample with lower roughness. The total resistance for each device was calculated, as depicted in **Supplementary Fig. 32**. A linear fitting analysis was performed, shown as the dashed line in **Supplementary Fig. 32**. The intercept of the linear fitting equals two times of contact resistance, as the slope equals to the resistivity over the width of the metal pad. In this case, the mobility of this device using TLM is modulated from 26.76 $cm^2\ V^{-1}\ s^{-1}$ to 34.57 $cm^2\ V^{-1}\ s^{-1}$ according to the equation as follows: $\mu = \frac{L}{qnC_{ox}R_{channel}}$, where μ is the mobility, L is the channel length, q is the elementary charge (approximately 1.602×10^{-19} C), n is the electron concentration, C_{ox} is the oxide capacitance per unit area. $R_{channel}$ is the intrinsic channel resistance.

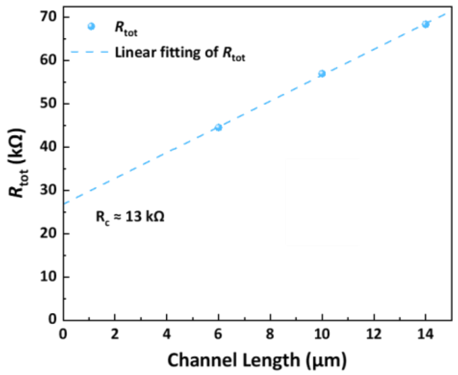

Supplementary Fig. 32 | Total resistance of channel and contact and its linear fitting against channel length. The total resistance of the channel and contact increases as the channel length increases. The intercept is 26 kΩ, two times the contact resistance, which means the contact resistance is 13 kΩ.

Section 12. Methodology for motion detection and recognition

The image is captured by the traditional CMOS sensor and the motion detection is done based on data from the whole 2D array (18 by 18) by simulation on MATLAB. The simulation of motion detection is implemented based on inter-frame differential computation in the following process: first, we experimentally record the negative photocurrent and the positive photocurrent of each device in an array scaled 18 by 18, and then transfer them to weights in the mapping matrix. Next, we set a suitable time interval Δt based on the motion pattern of the trolleys and extract the two frames (t_1, $t_1+\Delta t$) at the interval Δt, as shown in **Fig. 4b** in the manuscript. The prior frame pixels are mapped by the positive mapping matrix and stored, due to the non-volatile photoconductive memory properties. The latter frame pixels are mapped by the negative mapping matrix and the memorized result is combined with the mapping result of the previous frame. After obtaining the inter-frame sum, a threshold is defined to help differentiate the summed data. Finally, we reconstruct the classified data and transform it into a detected image. **Supplementary Fig. 33** shows the simulated results in the dark environment.

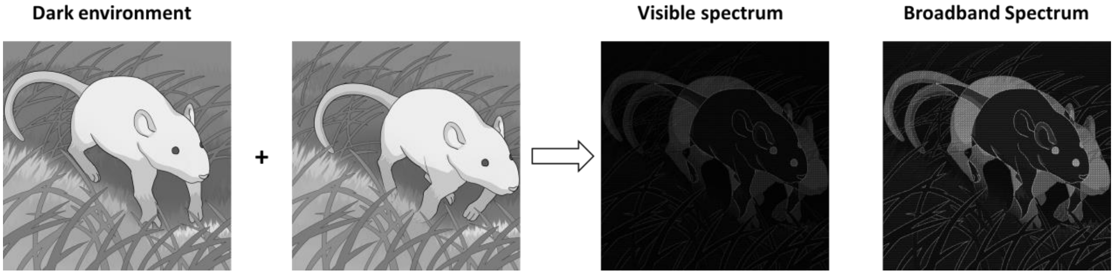

Supplementary Fig. 33 | Motion detection and recognition in dark environment. The motion detection and image recognition system was tested on a motion mouse in the dark environment using visible and broadband spectrum stimulation, which showed a significant improvement with using the broadband spectrum stimulation.

Section 13. Implementation in ANN

As our device integrates the functions of memory, sensing, and tunable photoconductivity, a simulation of an artificial neural network (ANN) is performed. **Supplementary Fig. 34** shows the perception flow of the Convolutional Neural Network (CNN) and the accuracy achieved at each training epoch based on the MNIST dataset.

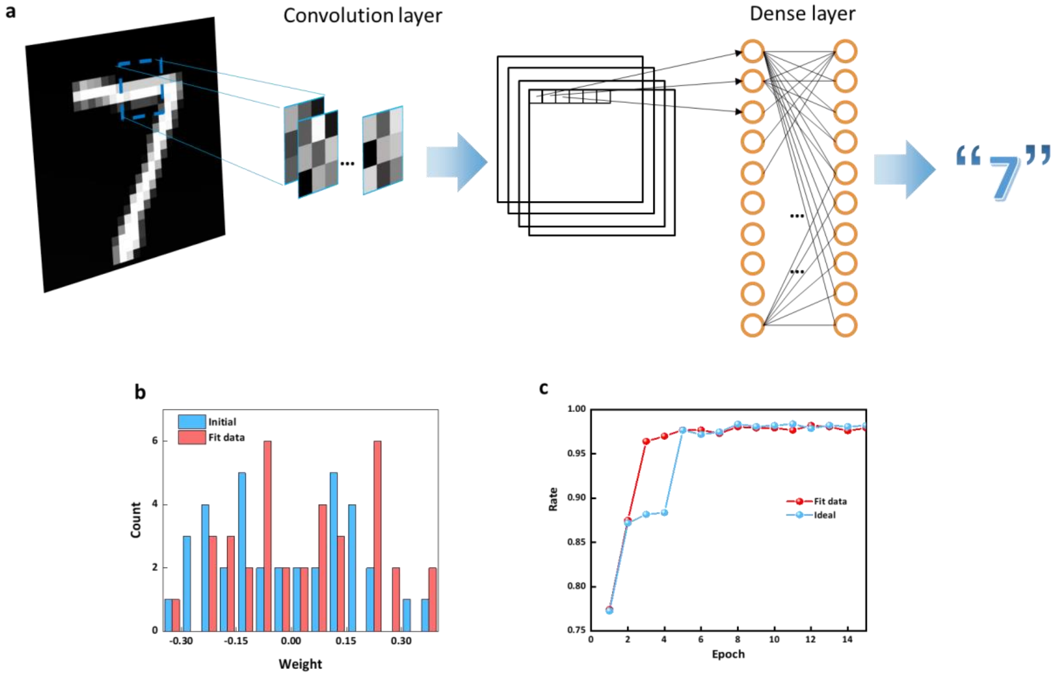

Supplementary Fig. 34 | Implementation of ANN using the RAO array. a, the perception flow of ANN performed based on the RAO array. **b,** The initial weight distribution and the weight distribution after training. **c,** Accuracy comparison of the ideal device and real device.

Section 14. Table of advanced optoelectronic devices

Materials	Number of pixels	Spectral range	Memory characteristic	Photoresponse mode	Moving detection	Ref.
Monolayer MoS_2	18*18	Visible-NIR	Non-volatile	Positive and negative	YES	Our work
BP/Al_2O_3/WSe_2/ h-BN heterostructure	3*3	Visible	Non-volatile	Positive and negative	YES	[7]
2D-perovskite	1*1	Visible	Non-volatile	Positive and negative	YES	[8]
WSe_2 homojunction	3*3	Visible	/	Positive and negative	NO	[9]
WSe_2 homojunction	3*9	Visible	Non-volatile	Positive	NO	[10]
Bilayer MoS_2 phototransistors	8*8	Visible	Volatile	Positive and negative	NO	[11]
Monolayer MoS_2	30*30	Visible	/	Positive	NO	[12]
Monolayer MoS_2	7*7	Visible	Volatile	Positive	NO	[13]
Doped Si	3*3	Visible-NIR	Non-volatile	Positive and negative	YES	[14]
MoS_2 photosensor	20*20	Visible	Volatile	Positive	YES	[15]

Supplementary Table 2 | Comparison of advanced optoelectronic devices.

Reference

1. Ng, H. K. et al. Improving carrier mobility in two-dimensional semiconductors with rippled materials. *Nat. Electron.* **5,** 489-496 (2022).
2. Kresse, G. & Furthmüller, J. Efficient iterative schemes for ab initio total-energy calculations using a plane-wave basis set. *Phys. Rev. B* **54,** 11169-11186 (1996).
3. Kresse, G. & Furthmüller, J. Efficiency of ab-initio total energy calculations for metals and semiconductors using a plane-wave basis set. *Comput. Mater. Sci.* **6,** 15-50 (1996).
4. Blöchl, P. E. Projector augmented-wave method. *Phys. Rev. B* **50,** 17953-17979 (1994).
5. Perdew, J. P., Burke, K. & Ernzerhof, M. Generalized Gradient Approximation Made Simple. *Phys. Rev. Lett.* **77,** 3865-3868 (1996).
6. Cheng, L. & Liu, Y. What Limits the Intrinsic Mobility of Electrons and Holes in Two Dimensional Metal Dichalcogenides? *J. Am. Chem. Soc.* **140,** 17895-17900 (2018).
7. Zhang, Z., Wang, S., Liu, C., Xie, R., Hu, W. & Zhou, P. All-in-one two-dimensional retinomorphic hardware device for motion detection and recognition. *Nat. Nanotechnol.* **17,** 27-32 (2022).
8. Lai, H. et al. Photoinduced Multi-Bit Nonvolatile Memory Based on a van der Waals Heterostructure with a 2D-Perovskite Floating Gate. *Adv. Mater.* **34,** 2110278 (2022).
9. Wang, C.-Y. et al. Gate-tunable van der Waals heterostructure for reconfigurable neural network vision sensor. *Sci. Adv.* **6,** eaba6173 (2020).
10. Mennel, L., Symonowicz, J., Wachter, S., Polyushkin, D. K., Molina-Mendoza, A. J. & Mueller, T. Ultrafast machine vision with 2D material neural network image sensors. *Nature* **579,** 62-66 (2020).
11. Liao, F. et al. Bioinspired in-sensor visual adaptation for accurate perception. *Nature Electronics* **5,** 84-91 (2022).
12. Dodda, A. et al. Active pixel sensor matrix based on monolayer MoS2 phototransistor array. *Nature Materials* **21,** 1379-1387 (2022).
13. Subbulakshmi Radhakrishnan, S., Dodda, A. & Das, S. An All-in-One Bioinspired Neural Network. *ACS Nano* **16,** 20100-20115 (2022).
14. Jang, H. et al. In-sensor optoelectronic computing using electrostatically doped silicon. *Nature Electronics* **5,** 519-525 (2022).
15. Chen, J. et al. Optoelectronic graded neurons for bioinspired in-sensor motion perception. Nat. Nanotechnol. 1-7 (2023), https://doi.org/10.1038/s41565-023-01379-2.
